# Evaluation of Antinociceptive Activity of Ethanol Extract of Leaves of *Adenanthera pavonina*

**DOI:** 10.1155/2015/412497

**Published:** 2015-08-04

**Authors:** Md. Moniruzzaman, Ambia Khatun, Mohammad Zafar Imam

**Affiliations:** ^1^College of Pharmacy, Dongguk University, Goyang 410-820, Republic of Korea; ^2^Department of Pharmacy, Stamford University Bangladesh, 51 Siddeswari Road, Dhaka 1217, Bangladesh

## Abstract

*Adenanthera pavonina* is a deciduous tree commonly used in the traditional medicine to treat inflammation and rheumatism. The aim of this study was to evaluate the antinociceptive activity of ethanol extract of leaves of *A. pavonina* (EEAP). EEAP was investigated using various nociceptive models induced thermally or chemically in mice including hot plate and tail immersion test, acetic acid-induced writhing, and glutamate- and formalin-induced licking tests at the doses of 50, 100, and 200 mg/kg body weight (p.o.). In addition, to assess the possible mechanisms, involvement of opioid system was verified using naloxone (2 mg/kg) and cyclic guanosine monophosphate (cGMP) signaling pathway by methylene blue (MB; 20 mg/kg). The results have demonstrated that EEAP produced a significant and dose-dependent increment in the hot plate latency and tail withdrawal time. It also reduced the number of abdominal constrictions and paw lickings induced by acetic acid and glutamate respectively. EEAP inhibited the nociceptive responses in both phases of formalin test. Besides, the reversal effects of naloxone indicated the association of opioid receptors on the exertion of EEAP action centrally. Moreover, the enhancement of writhing inhibitory activity by MB suggests the possible involvement of cGMP pathway in EEAP-mediated antinociception. These results prove the antinociceptive activity of the leaves of *A. pavonina* and support the traditional use of this plant.

## 1. Introduction

Inflammation is one of the most common physiological events that lead to chronic pain in response to tissue injury. It causes a consecutive change in several cellular components including neurotrophic factors, neuropeptides, prostanoids, and kinins which are able to conduct and amplify the nociceptive perception [1]. The changes in these cellular components have also been reported to generate and liberate different inflammatory mediators such as cytokines and chemokines by the immune or central nervous system cells, which cause the sensitization of the peripheral nociceptors [2]. However, the long term use of the currently available therapies to combat pain and inflammation tends to have serious side effects and low efficacy, especially for chronic diseases [3]. Thus, the development of agents that can control pain and inflammation with fewer side effects has been suggested to be a promising therapeutic approach to treat different painful conditions.

*Adenanthera pavonina* Linn. (family: Leguminosae-Mimosaceae), a deciduous tree commonly known as “Ranjan” in Bangladesh, is an important medicinal plant found in tropical Asia, western and eastern Africa, and most islands of both the Pacific and Caribbean regions. The leaf of this plant has long been used in the traditional medicine system against a wide range of diseases including inflammation and rheumatism [4, 5]. Researchers have isolated several methoxy flavonol glycosides, such as kaempferol-3-*O*-*α*-dirhamnopyranosyl-(1′′′ → 2′′, 1′′′′ → 6′′)-*β*-glucopyranoside, quercetin 3-*O*-*α*-dirhamnopyranosyl-(1′′′ → 2′′, 1′′′′ → 6′′)-*β*-glucopyranoside-4′-methoxy, isovitexin, quercetin-3-*O*-rhamnopyranosyl(1′′′ → 2′′)-*β*-glucopyranoside, quercetin-3-*O*-*β*-glucopranoside-4′-*O*-rhamnopyranoside, kaempferol-3-*O*-*α*-rhamnopyranosyl(1′′′ → 2′′)-*β*-glucopyranoside, quercetin-3-*O*-rhamnopyranosyl(1′′′ → 4′′)-*β*-glucopyranoside, quercetin-3-*O*-*β*-glucopyranoside, kaempferol, and quercetin from the extract of the leaves of* A. pavonina* [6]. Besides, several stigmasterol glucosides including octacosanol, dulcitol, glucosides of *β*-sitosterol, and stigmasterol have also been reported to be present in the leaves of this plant [7]. Pharmacological studies of the plant showed that the seeds of this plant possess anticonvulsant and central nervous system depressant, analgesic, and anti-inflammatory activity [8, 9]. But, so far, there is no report demonstrating the antinociceptive activity of the leaves of* A. pavonina* which prompted us to design our present study to evaluate the effectiveness of ethanol extract of this plant leaves in different nociceptive models and investigate the possible mechanism(s) involved in this effect.

## 2. Materials and Methods

### 2.1. Plant Material and Extraction

*A. pavonina* leaves were collected from Dhaka, Bangladesh, in January 2013. The samples were then recognized by Bushra Khan, Principal Scientific Officer of Bangladesh National Herbarium, Mirpur, Dhaka, Bangladesh. A voucher specimen (DACB: 37941) has been deposited to the Herbarium for further reference. Powdered dried leaves (250 g) were macerated with 350 mL of ethanol and subjected to occasional stirring at 25 ± 2°C for 3 days. Then the extract was collected and the solvent was completely removed by rotary evaporator. 11.52 g extract (yield 4.61% w/w) was obtained which was then used for all of the experimental studies.

### 2.2. Phytochemical Analyses

The crude extract of* A. pavonina* was assessed qualitatively to detect the presence of different phytochemicals such as carbohydrates, saponins, flavonoids, tannins, alkaloids, glycosides, glucosides, reducing sugars, proteins, gums, and steroids following standard procedures [4].

### 2.3. Chemicals and Drugs

Morphine sulphate was obtained from Gonoshasthaya Pharmaceuticals Ltd. (Savar, Bangladesh) and diclofenac sodium was obtained from Square Pharmaceuticals Ltd. (Dhaka, Bangladesh). Naloxone was purchased from Hameln Pharmaceuticals GmbH (Hameln, Germany). Acetic acid, L-glutamic acid, formalin, methylene blue, ethanol, and 99% dimethyl sulfoxide (DMSO) were procured from Merck (Darmstadt, Germany).

### 2.4. Animals

In the entire set of experiment, Swiss albino mice (20–25 g) of either sex were used. The animals were purchased from Animal Resources Branch of the International Center for Diarrheal Disease Research, Bangladesh (icddr,b). The animals were kept in standard laboratory conditions (relative humidity: 55–60%; room temperature: 25 ± 2°C; 12 h light/12 h dark cycle) and were provided with standard diet (icddr,b formulated) and water* ad libitum*. The animals were kept in the laboratory 14 days prior to the experiments so they can acclimatize to the laboratory environment. The animals were kept fasting overnight before the experiments to avoid any possible food-extract/drug interaction. All the experimental animals were treated following the “Ethical Principles and Guidelines for Scientific Experiments on Animals” (1995) formulated by The Swiss Academy of Medical Sciences and the Swiss Academy of Sciences. All experimental steps conducted in this study were permitted by the Institutional Ethics Committee of Stamford University Bangladesh.

### 2.5. Drugs and Treatments

Morphine sulphate (5 mg/kg) was employed in hot plate, tail immersion, and formalin tests and diclofenac sodium (10 mg/kg) was used in writhing and glutamate-induced licking tests as standard drug. These drugs were administered intraperitoneally (i.p.) 15 min before the induction of nociception. In both chemical- and heat-induced pain models, EEAP was administered orally 30 min prior to the experiments at the doses of 50, 100, and 200 mg/kg, where the animals in control group received DMSO (vehicle; 0.1 mL/mouse, p.o.).

### 2.6. Acute Toxicity Test

Animals were randomly assigned to different groups containing 5 mice in each group. EEAP was administered to the animals orally at the doses of 500, 1000, and 2000 mg/kg. The mice were allowed food and water* ad libitum* and all animals were observed for abnormal behaviors, allergic symptoms, and mortality for the next 72 h [10].

### 2.7. Antinociceptive Analysis

#### 2.7.1. Hot Plate Test

The hot plate test was performed according to previously described method [11]. The animals were selected 24 h prior to the experiment according to their responses such as forepaw licking, paw withdrawal, or jumping within 15 s of thermal stress. Then they were treated with EEAP or morphine as mentioned above and placed on Eddy's hot plate (Kshitij Innovations, Haryana, India) kept at a temperature of 50 ± 0.5°C. A cut-off time was maintained for 20 s to avoid paw tissue damage of the animals. The latency was then recorded following their behavior in the hot plate at 30, 60, 90, and 120 min after the treatment. Finally, the percentage of the maximal possible effect (%MPE) was calculated using the following equation:

(1) %MPE=Postdrug latency−Predrug latencyCut-off time−Predrug latency×100.

#### 2.7.2. Tail Immersion Test

This experiment was done based on the previous observation demonstrating that morphine like analgesics prolong the tail withdrawal latency from hot water in mice [12]. Mice that showed tail withdrawal time between 1.5 and 3.5 s were selected for this experiment and the pretreatment latency was recorded. Then the animals were pretreated with morphine or EEAP and one to two cm of their tail was immersed in the warm water with constant temperature of 54 ± 0.5°C. The time between tail submersion and tail deflection was recorded at 30, 60, 90, and 120 min after the treatment with standard drug or extract. A cut-off time of 20 s was maintained to avoid tail tissue damage in the animals. Then %MPE was calculated using the same formula employed in hot plate test.

#### 2.7.3. Acetic Acid-Induced Writhing Test

The animals were treated with standard drug or EEAP or vehicle and then the writhing was induced with injection of 0.6% acetic acid 15 min after drug or 30 min after EEAP administration. Five minutes after acetic acid administration, the mice were observed and the writhing number was counted for 30 min as described previously [13]. The incidences of contractions of the abdomen, elongation of the body, twisting of the trunk, and/or pelvis ending were considered as complete writhing.

#### 2.7.4. Glutamate-Induced Nociception

10 *μ*M of glutamate was injected into the ventral surface of the right hind paw of the mice 30 min after EEAP treatment or 15 min after injection of diclofenac sodium. The animals were then observed for 15 min following glutamate injection and the licking number of its injected paw was recorded as an indication of nociception [14].

#### 2.7.5. Formalin-Induced Nociception

60 min after EEAP or 15 min after treatment with morphine, 20 *μ*L of 2.5% formalin solution was injected into the subplantar region of the right hind paw of the mice. The licking or biting of the injected paw was then recorded from 0–5 min as neurogenic phase and 15–30 min for inflammatory phase [15, 16].

### 2.8. Analysis of the Possible Mechanism of Action of EEAP

#### 2.8.1. Involvement of Opioid System

The possible involvement of the opioid receptors system in the antinociceptive effect of EEAP was examined by injecting naloxone hydrochloride (2 mg/kg i.p.), a nonspecific opioid receptor antagonist, 15 min prior to the administration of either morphine or EEAP. The hot plate and tail immersion latencies were sequentially measured at 30, 60, 90, and 120 min after treatment of morphine or EEAP [17].

#### 2.8.2. Involvement of Cyclic Guanosine Monophosphate (cGMP) Pathway

To validate the possible participation of cGMP pathway in the antinociceptive action caused by EEAP, the mice were pretreated with a nonspecific inhibitor of NO/guanylyl cyclase (MB) at the dose of 20 mg/kg, i.p. 15 min before the administration of EEAP. Then the nociceptive responses against 0.6% acetic acid injection were observed for 30 min, starting from 5 min after injection. The numbers of abdominal writhing were considered as the scoring of pain behavior [18].

### 2.9. Statistical Analysis

The results are expressed as mean ± SEM. The statistical analysis was performed by one-way analysis of variance (ANOVA) followed by Dunnett's or Bonferroni's post hoc test as appropriate, using SPSS 11.5 software. Differences between groups were considered significant at *p* < 0.05.

## 3. Results

### 3.1. Phytochemical Screening

Preliminary phytochemical screening of the crude ethanol extract of the leaves of* A. pavonina* confirmed the presence of alkaloids, carbohydrates, proteins, flavonoids, glycosides, saponins, steroids, and tannins.

### 3.2. Acute Toxicity Test

The oral administration of EEAP at the doses of 500–2000 mg/kg did not produce any abnormal behavior of the animals. The same dose of EEAP also did not cause any allergic manifestation or mortality during the observation period of 72 h after administration. Therefore, it is possible that EEAP may not be toxic at all the doses used in this study up to 2000 mg/kg.

### 3.3. Hot Plate Test

As shown in Table 1, all the doses of EEAP exhibited antinociceptive activity but with varying degree in the hot plate algesiometer-based evaluation of nociception in mice. Oral administration of EEAP significantly increased the latency period at 100 and 200 mg/kg (*p* < 0.01) doses at 60–120 min observation period. As expected, morphine at 5 mg/kg demonstrated a significant antinociceptive effect compared to control (*p* < 0.001). Naloxone at 2 mg/kg dose significantly reversed the antinociceptive effect of morphine (*p* < 0.001) or EEAP at 100 and 200 mg/kg doses (*p* < 0.05).

### 3.4. Tail Immersion Test

In the tail immersion test, EEAP showed marked antinociceptive activity in a dose-dependent manner (Table 2). More specifically, at 60 min after oral administration, EEAP at both 100 and 200 mg/kg doses significantly delayed (*p* < 0.01) the reaction time in response to a nociceptive stimulus. Morphine, the reference drug, also exhibited strong antinociceptive activity where naloxone significantly attenuated the antinociceptive effect of morphine (*p* < 0.01) as well as EEAP at 100 and 200 mg/kg doses (*p* < 0.05), in parallel with the findings of the hot plate test.

### 3.5. Acetic Acid-Induced Writhing Test

**Figure 1:**
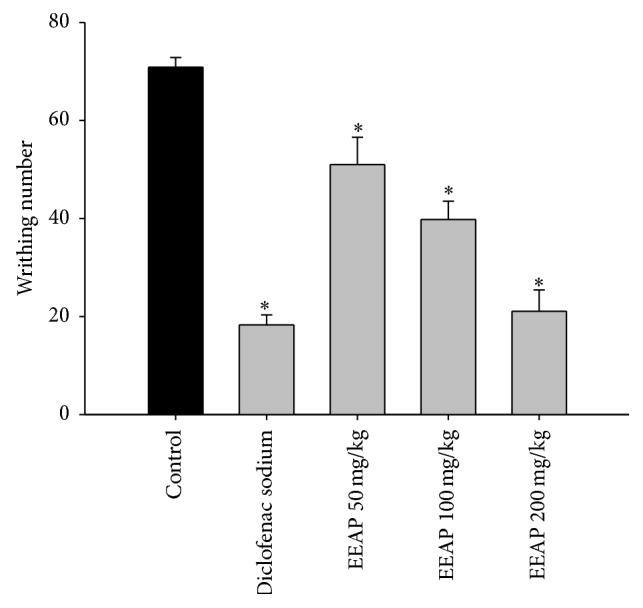
Antinociceptive effect of EEAP (50, 100, and 200 mg/kg, p.o.) and diclofenac sodium (10 mg/kg, i.p.) in the nociception induced by acetic acid in mice. Statistical analysis was performed using one-way ANOVA followed by Dunnett's post hoc test. The results are expressed as mean ± SEM (*n* = 5). ^*∗*^ *p* < 0.01 compared to control.

The extent of writhing in mice induced by administration of 0.6% of acetic acid was significantly suppressed (*p* < 0.01) by all of the doses of EEAP (Figure 1). This suppression is comparable to the writhing inhibitory effect of diclofenac sodium (74.19%) used as a reference drug.

### 3.6. Glutamate-Induced Nociception

**Figure 2:**
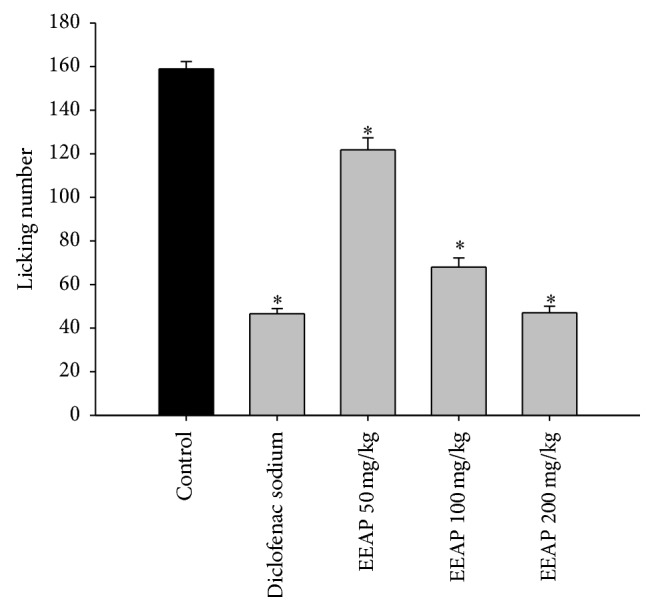
Antinociceptive effect of EEAP (50, 100, and 200 mg/kg, p.o.) and diclofenac sodium (10 mg/kg, i.p.) in the nociception induced by glutamate in mice. Statistical analysis was performed using one-way ANOVA followed by Dunnett's post hoc test. The results are expressed as mean ± SEM (*n* = 5). ^*∗*^ *p* < 0.001 compared to control.

The oral administration of EEAP (50, 100, and 200 mg/kg) caused a significant inhibition of the glutamate-induced nociception in a dose-dependent manner (Figure 2). The reference drug diclofenac sodium also produced significant antinociceptive effect (*p* < 0.001).

### 3.7. Formalin-Induced Nociception

**Figure 3:**
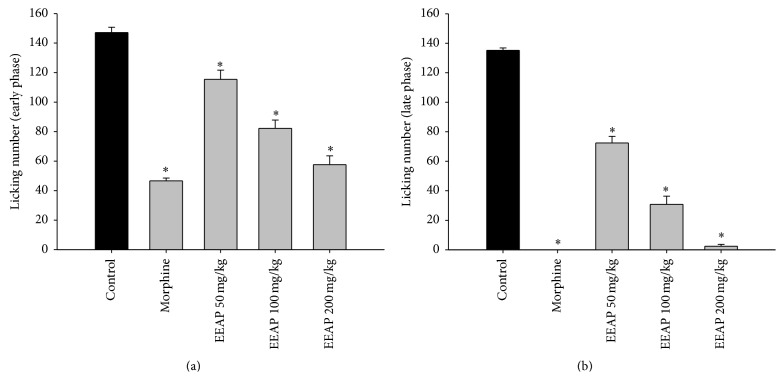
Response frequencies of the right hind paws injected with 2.5% formalin (20 *μ*M/paw) in control group and in mice treated with EEAP (50–200 mg/kg, p.o.) or diclofenac sodium (10 mg/kg, i.p.) in early phase (a) and in late phase (b). Each group represents the mean ± SEM (*n* = 5). Statistical analysis was performed using one-way ANOVA followed by Dunnett's post hoc test. ^*∗*^ *p* < 0.001 compared to control.

In both phases of the formalin test, EEAP caused a dose-dependent inhibition of the licking number induced by formalin (Figure 3). The effect is statistically significant (*p* < 0.001) with all of the experimental doses, where 60.87% of licking inhibition in the first phase and 98.22% inhibition in second phase were observed with the dose of 200 mg/kg of EEAP.

### 3.8. Involvement of Cyclic Guanosine Monophosphate (cGMP) Pathway

The present study evaluated the impact of MB treatments (20 mg/kg) on the antinociceptive activity of EEAP at 50, 100, and 200 mg/kg doses. Treatment with EEAP or MB alone significantly inhibited acetic acid-induced abdominal writhing (Table 3). The extent of EEAP-induced antinociception effect was significantly (*p* < 0.05) enhanced when EEAP was cotreated with MB.

## 4. Discussion

This study evaluated the effects of the crude ethanol extract of* A. pavonina*, using several in vivo models of nociception in rodents. The results demonstrated that oral administration of EEAP significantly reduced the nociceptive responses in a dose-dependent manner.

Hot plate and tail immersion tests on mice were used to evaluate the effect of extract against thermal stimuli. These tests are widely used to investigate centrally acting analgesic dugs which delays the response against heat-induced pain thresholds [19, 20]. More specifically, the tail immersion and the hot plate tests serve as a popular tool to monitor the spinal and supraspinal reflexes, respectively [21]. Further elaborative studies have revealed that *μ*2- and *δ*-opioid receptors are involved in spinal mechanism, while *μ*1/*μ*2-opioid receptors are speculated to be primarily associated with supraspinal analgesia [22, 23]. In this study, EEAP at both 100 and 200 mg/kg doses significantly increased the time latency in hot plate test revealing the central antinociceptive activity of EEAP. This effect was parallel to the impact of EEAP in the tail immersion test. To further investigate possible antinociceptive mechanism(s) of EEAP action, the effect of naloxone, a nonselective opioid receptor antagonist, was examined against the antinociceptive effect of EEAP. The results revealed that naloxone reverses the antinociceptive effect of EEAP to some extent which impressed us to conceive that the opioid receptors may influence the central antinociceptive effect of EEAP through spinal and supraspinal mechanisms.

The acetic acid-induced writhing test is a relatively simple and rapid one but is considered with low specificity. In this experiment, acetic acid acts as a potent inducer of writhing syndrome and causes algesia by increasing the level of proinflammatory mediators cyclooxygenase (COX), lipoxygenase (LOX), prostaglandins (PGs), histamine, serotonin, bradykinin, substance P, IL-1*β*, IL-8, and TNF-*α* in the peripheral tissue fluid, which then excite the peripheral nociceptive nerve endings [24, 25] resulting in inflammatory pain [26]. The results demonstrated that EEAP at the doses of 50, 100, and 200 mg/kg significantly reduced the number of writhing episodes in mice, indicating the inhibition of acetic acid-induced visceral nociception. This probably suggests that the inflammatory pathways might be the target of EEAP and that inhibitory action of EEAP may be due to the downregulation of synthesis, release, or retardation of action of the above mentioned endogenous substances or cytokines leading to an interference with the transduction of signals mediated through primary afferent nociceptors [27].

Oral administration of EEAP also significantly inhibited the noxious stimuli induced by L-glutamic acid in a dose-dependent manner. It has been reported that, among different excitatory amino acids, glutamate and aspartate play an important role in pain perception. In this study, intraplantar injection of glutamate produced the nociceptive response in mice. This phenomenon has been shown to be mediated through the action of glutamate on both N-methyl-D-aspartate (NMDA) and non-NMDA receptors in peripheral, spinal, and supraspinal sites [14]. Additionally, glutamate is also known to cause the release of proinflammatory mediators like nitric oxide (NO) and NO-related substances in both central and peripheral nervous systems [28]. Taking the above in consideration, it is conceivable that the antinociceptive activity of EEAP may be associated with its interaction with the glutamatergic system.

Finally, this study evaluated the antinociceptive activity of EEAP using formalin test. This test is a widely used model for studying pain and analgesia. Intraplantar injection of formalin in the paw was found to produce a biphasic nociception [29]. The sensation of this intensive pain seems to be due to the activation of primary afferent sensory neurons via specific and direct actions of the endogenous proinflammatory agents and cytokines on the Transient Receptor Potential Vanilloid-1 (TRPA-1), a member of the Transient Receptor Potential family (TRP) of cation channels that is highly expressed by a subset of C-Fiber nociceptors [30]. This is also in agreement with a previous study revealing that a biphasic release of PGE2 plays a vital role in formalin-induced nociceptive behavior [31]. In this study, EEAP caused significant inhibition of formalin-induced nociception in mice in a dose-dependent manner, both in neurogenic phase (early phase) and inflammatory phase (late phase), prompting us to consider that the antinociceptive activity of EEAP could be related to the suppression of proinflammatory mediators in the cyclooxygenase pathway.

To further investigate whether the antinociceptive action of EEAP involves the cGMP pathway, MB, a nonspecific inhibitor of NO/guanylyl cyclase, was used. NO, an essential bioregulatory molecule, has been shown to increase the level of cGMP by activating soluble guanylyl cyclase (sGC), leading to a wide range of physiological consequences including pain and analgesia [32]. It has been reported that the cGMP can act on the ion channels directly or may activate protein kinases and phosphodiesterases [33]. The results demonstrated that pretreatment with MB, which inhibits peripheral NO production and sGC activation [32], significantly reduced the acetic acid-induced pain perception and also enhanced the antinociceptive activity of EEAP. This suggests that EEAP probably involves the NO-cGMP pathway in its antinociceptive mechanism in the prevailed experimental conditions.

It has been reported that the natural antioxidants mainly derive from plants in the form of phenolic compounds such as flavonoid, phenolic acids, and tocopherols [34]. Phytochemical analysis has revealed that the crude ethanol extract of* A. pavonina* leaf contains alkaloids, carbohydrates, proteins, flavonoids, glycosides, saponins, steroids, and tannins. These compounds may contribute to the antinociceptive activity of EEAP. It has been reported that flavonoids interact directly with the cyclooxygenase pathway, resulting in the inhibitions of PGs [35]. The flavonoids can also suppress the increased level of intracellular Ca^2+^ ion and the release of proinflammatory mediators in a dose-dependent manner [36]. Besides, tannins, saponins, and glycosides are also found to elicit analgesic and anti-inflammatory activities which are mediated through the inhibition of cyclooxygenases [37, 38]. Taken together, it is conceivable that the observed antinociceptive effects of EEAP may be due to the presence of the above mentioned phytochemicals in the extract.

## 5. Conclusions

In conclusion, the present study demonstrated the central and peripheral antinociceptive activity of ethanol extract of* A. pavonina* leaves against different animal models of acute nociception that can be related to the ethnomedicinal use of this plant leaves in treatment of different painful conditions. This study also suggests that the opioid receptors and cGMP pathway may contribute to the observed antinociceptive actions of EEAP. So, this multiplicity of mechanisms exhibited by the EEAP opens a great opportunity to develop multitarget drug candidates to treat pain and inflammation.

## Figures and Tables

**Table 1:** Antinociceptive effect of ethanol extract of leaves of *A. pavonina* and morphine and reversal effect of naloxone in hot plate test.

Treatment (mg/Kg)	Response time (s) (% MPE)				
	Pretreatment	30 min	60 min	90 min	120 min
Control (0.1 mL/mouse)	6.05 ± 0.19	6.24 ± 0.39	6.82 ± 021	7.46 ± 0.16	7.86 ± 0.28
Morphine (5)	6.21 ± 0.19	12.84 ± 0.84^*∗*^ (48.09)	15.03 ± 1.21^*∗*^ (63.99)	15.59 ± 0.61^*∗*^ (68.01)	15.83 ± 1.31^*∗*^ (69.75)
EEAP (50)	5.72 ± 0.74	7.84 ± 0.63 (14.90)	9.11 ± 0.65 (23.77)	9.97 ± 0.63 (29.81)	10.40 ± 0.31 (32.78)
EEAP (100)	6.31 ± 0.34	8.22 ± 0.41 (13.96)	10.58 ± 0.32^*∗∗*^ (31.17)	11.45 ± 1.00^*∗∗*^ (37.53)	11.58 ± 1.13 (38.45)
EEAP (200)	5.83 ± 0.66	8.85 ± 0.60 (21.35)	11.94 ± 0.10^*∗∗*^ (43.11)	12.96 ± 0.81^*∗∗*^ (50.32)	14.31 ± 0.52^*∗∗*^ (59.84)
NLX (2) + control (0.1 mL/mouse)	5.85 ± 0.43	5.92 ± 0.30	6.08 ± 0.33	6.32 ± 0.34	7.06 ± 0.22
NLX (2) + morphine (5)	6.01 ± 0.55	6.79 ± 0.72^a^ (5.57)	7.94 ± 0.56^a^ (13.81)	8.39 ± 0.58^a^ (17.01)	10.27 ± 1.48^a^ (30.47)
NLX (2) + EEAP (50)	5.92 ± 0.32	6.84 ± 0.37 (6.57)	7.10 ± 0.33 (8.44)	8.34 ± 0.84 (17.24)	8.81 ± 0.57 (20.53)
NLX (2) + EEAP (100)	5.89 ± 0.42	7.35 ± 0.44 (10.35)	7.55 ± 0.32^b^ (11.76)	8.46 ± 0.56 (18.23)	9.08 ± 0.56 (22.58)
NLX (2) + EEAP (200)	6.03 ± 0.42	7.54 ± 0.59 (10.82)	8.60 ± 0.32^c^ (18.40)	9.45 ± 0.50^c^ (24.52)	10.95 ± 0.69 (35.26)

Each value is presented as the mean ± SEM (*n* = 5). EEAP = ethanol extract of *A. pavonina*; NLX = naloxone; ^*∗*^ *p* < 0.001 compared with the control group (Dunnett's test); ^*∗∗*^ *p* < 0.01 compared with the control group (Dunnett's test); ^a^ *p* < 0.001 compared with the morphine group (Bonferroni's test); ^b^ *p* < 0.05 compared with the EEAP 100 mg/kg group (Bonferroni's test); ^c^ *p* < 0.05 compared with the EEAP 200 mg/kg group (Bonferroni's test).

**Table 2:** Antinociceptive effect of ethanol extract of leaves of *A. pavonina* and morphine and reversal effect of naloxone in tail immersion test.

Treatment (mg/Kg)	Response time (s) (% MPE)				
	Pretreatment	30 min	60 min	90 min	120 min
Control (0.1 mL/mouse)	1.88 ± 0.22	2.16 ± 0.39	2.38 ± 0.15	2.56 ± 0.18	2.74 ± 0.18
Morphine (5)	1.74 ± 0.05	3.12 ± 0.30 (7.58)	3.93 ± 0.21^*∗*^ (11.98)	4.37 ± 0.27^*∗*^ (14.43)	4.48 ± 0.04^*∗*^ (15.01)
EEAP (50)	1.75 ± 0.11	2.33 ± 0.14 (3.21)	2.72 ± 0.22^*∗∗*^ (5.35)	2.96 ± 0.27^*∗∗*^ (6.64)	3.16 ± 0.16 (7.77)
EEAP (100)	1.94 ± 0.32	2.80 ± 0.15 (4.76)	3.28 ± 0.11^*∗∗*^ (7.41)	3.63 ± 0.13^*∗∗*^ (9.33)	3.78 ± 0.41^*∗∗*^ (10.15)
EEAP (200)	1.99 ± 0.20	2.95 ± 0.19 (5.36)	3.58 ± 0.17 (8.87)	4.09 ± 0.19 (11.67)	4.02 ± 0.22 (11.27)
NLX (2) + control (0.1 mL/mouse)	1.79 ± 0.05	1.99 ± 0.22	1.97 ± 0.14	2.11 ± 0.23	2.23 ± 0.22
NLX (2) + morphine (5)	1.65 ± 0.10	2.07 ± 0.17 (2.29)	2.78 ± 0.25^a^ (6.17)	2.91 ± 0.20^a^ (6.87)	3.24 ± 0.21^a^ (8.69)
NLX (2) + EEAP (50)	1.71 ± 0.16	2.14 ± 0.15 (2.36)	2.30 ± 0.16 (3.25)	2.46 ± 0.16 (4.10)	2.97 ± 0.27 (6.88)
NLX (2) + EEAP (100)	1.89 ± 0.35	2.31 ± 0.14 (2.31)	2.45 ± 0.13 (3.09)	2.54 ± 0.18^b^ (3.57)	3.20 ± 0.19 (7.21)
NLX (2) + EEAP (200)	1.65 ± 0.25	2.50 ± 0.28 (4.64)	2.66 ± 0.17^c^ (5.50)	3.08 ± 0.29 (7.80)	3.31 ± 0.18 (9.05)

Each value is presented as the mean ± SEM (*n* = 5). EEAP = ethanol extract of *A. pavonina*; NLX = naloxone; ^*∗*^ *p* < 0.001 compared with the control group (Dunnett's test); ^*∗∗*^ *p* < 0.01 compared with the control group (Dunnett's test); ^a^ *p* < 0.01 compared with the morphine group (Bonferroni's test); ^b^ *p* < 0.05 compared with the EEAP 100 mg/kg group (Bonferroni's test); ^c^ *p* < 0.05 compared with the EEAP 200 mg/kg group (Bonferroni's test).

**Table 3:** Effect of EEAP on involvement of cyclic guanosine monophosphate (cGMP) pathway.

Treatment (mg/Kg)	Writhing number (Mean ± SEM)	% Inhibition
Control (0.1 mL/mouse)	73.30 ± 2.76	
MB (20)	55.70 ± 4.94	24.01
EEAP (50)	49.20 ± 3.58^*∗*^	32.88
EEAP (100)	39.60 ± 4.21^*∗*^	45.98
EEAP (200)	23.60 ± 1.47^*∗*^	67.80
MB (20) + EEAP (50)	41.30 ± 2.31	43.66
MB (20) + EEAP (100)	28.90 ± 2.87	60.57
MB (20) + EEAP (200)	10.20 ± 0.82^a^	86.08

Values are presented as the mean ± SEM (*n* = 5). EEAP = ethanol extract of *A. pavonina*; MB = methylene blue; ^*∗*^ *p* < 0.001 compared with the control group (Dunnett's test); ^a^ *p* < 0.05 compared with the EEAP 200 mg/kg group (Bonferroni's test).
